# Oxygen Vacancy in Magnéli Phases and Its Effect on Thermoelectric Performances

**DOI:** 10.3390/nano15090684

**Published:** 2025-04-30

**Authors:** Zhou Guan, Chuangshi Feng, Hongquan Song, Lingxu Yang, Xin Wang, Huijun Liu, Jiawei Zhang, Fanqian Wei, Xin Yuan, Hengyong Yang, Yu Tang, Fuxiang Zhang

**Affiliations:** 1 Songshan Lake Materials Laboratory, Dongguan 523808, China; guanzhou@sslab.org.cn (Z.G.); fengchuangshi@sslab.org.cn (C.F.); 72404517@cityu-dg.edu.cn (X.W.); 2300451012@email.szu.edu.cn (J.Z.); 202421020135@mail.scut.edu.cn (F.W.); 21123653r@connect.polyu.hk (X.Y.); 202221003740@mail.scut.edu.cn (H.Y.); 22ytang@stu.edu.cn (Y.T.); 2 College of Physics and Telecommunication Engineering, Zhoukou Normal University, Zhoukou 466001, China; songhq@zknu.edu.cn; 3 School of Ocean Engineering, Guangzhou Maritime University, Guangzhou 510330, China; yanglingxu@sslab.org.cn

**Keywords:** Magnéli phase, oxygen vacancy, thermoelectric performance, single parabolic band

## Abstract

Magnéli phases exhibit significant potential for applications in electronic materials in energy conversion due to their high electrical conductivity and excellent thermal stability. In this study, single-phase Ti_n_O_2n−1_ (*n* = 4, 5, 6) bulk materials were successfully prepared by a combination of the carbothermal reduction of nano-sized rutile TiO_2_ and hot-press sintering methods. The relationships between the phase evolution, microstructural features, and thermoelectric performance were investigated systematically. Synchrotron X-ray diffraction (SXRD) and scanning electron microscopy (SEM) analyses revealed that the Ti_4_O_7_ and Ti_5_O_9_ materials had single-phase structures with high densities (relative density > 97%) and no obvious grain boundary holes or microcracks. We tested the thermoelectric properties of the Magnéli phases in the temperature range of 300–1100 K. The Magnéli phases exhibited a significant temperature dependence, with peak zT values of 0.17, 0.18, and 0.14 for Ti_4_O_7_, Ti_5_O_9_, and Ti_6_O_11_, respectively, at 1100 K. This variation in thermoelectric performance was mainly attributed to the synergistic effect of the oxygen vacancy concentration and the shear surface density on the carrier concentration and lattice thermal conductivity. Furthermore, the Fermi energy levels and electronic thermal conductivity of the Magnéli phases were calculated using the single parabolic band (SPB) model.

## 1. Introduction

In recent years, significant advancements have been achieved in the research of thermoelectric materials, driven by the global energy transition and the development of green technology. Conventional thermoelectric materials, such as SnSe, Bi_2_Te_3_, PbTe, and SiGe [1,2,3,4,5,6], exhibit a relatively high thermoelectric figure of merit (zT values). However, they face critical limitations, including environmental toxicity, degradations in high-temperature performance, and prohibitive manufacturing costs, which severely restrict their large-scale applications. For instance, PbTe tends to decompose at elevated temperatures, releasing toxic lead vapors, while the synthesis of SiGe alloys requires high-purity inert atmospheres, resulting in complex and costly processes. In response to these challenges, metal oxide thermoelectric materials, which combine environmental compatibility with excellent chemical stability, have emerged as a research focus. Taking perovskite-type SrTiO_3_ as an example, aliovalent doping with La^3+^ or Nb^5+^ enables precise carrier concentration control, demonstrating breakthrough potential, with zT values exceeding 0.3, and establishing a paradigm for oxide thermoelectric systems [7,8].

As another representative metal oxide, titanium dioxide (TiO_2_) has attracted considerable attention due to its exceptional chemical stability, low ecological toxicity, and abundant natural reserves [9]. However, the wide bandgap (~3.0 eV for rutile and ~3.2 eV for anatase) and ultralow electrical conductivity (<10^−3^ S·cm^−1^) of intrinsic TiO_2_ severely limit its thermoelectric performance [10,11,12,13,14]. To address these limitations, researchers have adopted dual optimization strategies: on the one hand, aliovalent doping with Nb^5+^ or Ta^5+^ enhances carrier concentration, boosting electrical conductivity to the order of 10² S·cm⁻¹ and achieving a zT value of 0.2 at 800 K [15,16,17,18]; on the other hand, constructing oxygen-vacancy-ordered Magnéli-phase titanium oxides (TiₙO_2n−1_ ) through periodic oxygen vacancy arrangements within shear planes simultaneously increases carrier concentration (~10^21^ cm^−3^) and reduces lattice thermal conductivity (κ_l_ < 1.8 W·m^−1^·K^−1^), revealing unique potential for thermoelectric regulation. Portehault et al. synthesized nanostructured Ti₄O₇ via sol–gel combined with spark plasma sintering (SPS), reducing κ_l_ to 1.0 W·m^−1^·K^−1^ through grain boundary scattering. However, the maximum zT value reached was only 0.08 at 1073 K [19]. Ti_5_O_9_, prepared by Pandey’s team using solid-state reaction and hot-pressing sintering, achieved a zT value of 0.3 at 1076 K [20]. Fan et al. reported a zT value of 0.16 at 764 K for Ti_9_O_17_ [21], while Wang et al. obtained zT = 0.32 at 973 K through a Ti_6_O_11_/Ti_7_O_13_ mixed-phase structure. Nevertheless [22], current research remains fragmented, lacking systematic analysis of the structure–property relationships in Ti_n_O_2n−1_ systems.

In this work, high-purity Ti_n_O_2n−1_ (*n* = 4, 5, 6) materials were synthesized through controlled titanium oxide reduction, and single-phase, high-density bulk samples (with relative densities of greater than 97%) were fabricated by using hot-pressure sintering. The electrical conductivity (σ), Seebeck coefficient (S), and thermal conductivity (κ) of these samples were characterized systematically over a wide temperature range (300–1100 K), and the zT values were calculated to evaluate the thermoelectric properties. Notably, we correlated and analyzed the measurement data using a single parabolic band model. This study aims to elucidate the quantitative correlation between oxygen vacancy ordering and thermoelectric parameters, providing an experimental basis for the rational design of Magnéli-phase materials.

## 2. Experimental Section

### 2.1. Preparation

**Figure 1:**
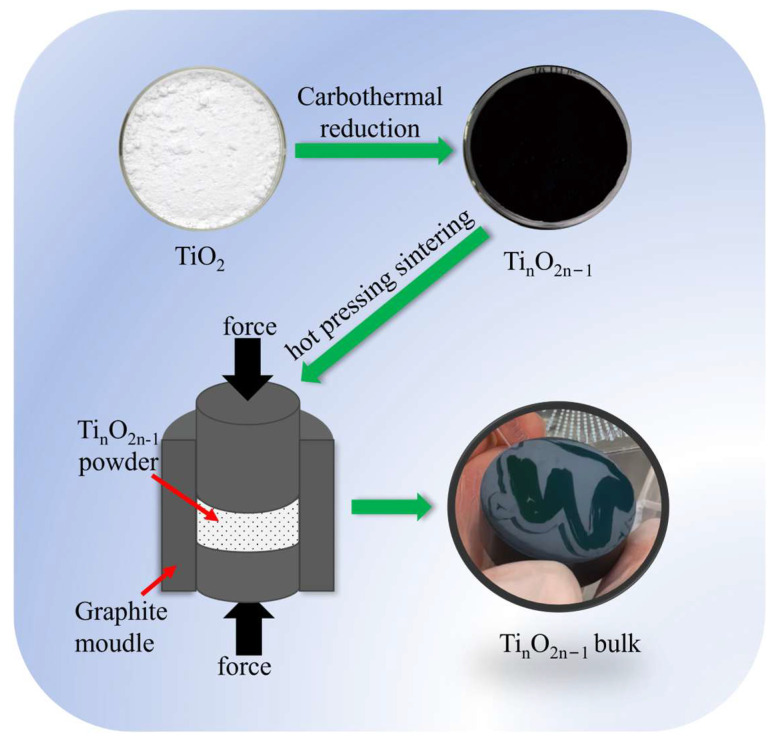
Schematic of the preparation of bulk Magnéli-phase Ti_n_O_2n−1_.

High-purity titanium dioxide powder with a nano-size rutile structure was subjected to a reduction reaction, and pure powder of Magnéli-phase Ti_n_O_2n−1_ (*n* = 4, 5, 6) was obtained. The powder was formed into high-densification bulks using a hot-press sintering method. A total of 120 g of powder was loaded into a graphite mold with a diameter of 60 mm and placed in a hot-press furnace. Before heating, the chamber was evacuated down to a pressure of 5 Pa, followed by purging with high-purity argon (99.999%). The above process was repeated three times to minimize the oxygen content in the chamber. The sample was sintered under a pressure of 30 MPa for 30 min at a heating rate of 10 °C per minute in a flowing argon atmosphere at 1200 °C. After cooling down to ambient temperature, the as-prepared samples were mechanically ground with SiC sandpapers to remove adhering graphite paper and then polished to obtain a bright surface (as shown in Figure 1). Finally, the samples were cut into two different sizes using a diamond wire cutter (STX-202A, KEJING, Shenyang, China): 3 × 3 × 10 mm (thermoelectric system test) and 10 × 10 × 1 mm (thermal conducting test).

### 2.2. Characterization

The crystal structure of the initial powder of the Magnéli-phase Ti_n_O_2n−1_ was characterized by synchrotron radiation diffraction measurement at the BL14B1 beamline of the Shanghai Synchrotron Radiation Facility with an incident X-ray wavelength of 0.6199 Å, and the hot-pressed bulk material was measured using a Rigaku (Tokyo, Japan) Miniflex-600 X-ray diffractor (copper target wavelength of 1.5169 Å). The microstructure was observed using scanning electron microscopy (SEM, ThermoFisher, Verios 5UC, Waltham, MA, USA) with electron backscatter diffraction (EBSD), respectively. The Seebeck coefficient and electrical conductivity were measured with commercial equipment (CTA3S/1150, Cryoall, Beijing, China) under a helium atmosphere. The thermal diffusivity of the Ti_n_O_2n−1_ was measured using a laser flash method (LFA 500, Linseis, Selb, Germany), and the heat capacity (*Cp*) of the samples was calculated by the Dulong–Petit law. The density was measured by the Archimedean method (SQP, Sartorius, Göttingen, Germany).

## 3. Results and Discussion

### 3.1. Characterization of Structure

**Figure 2:**
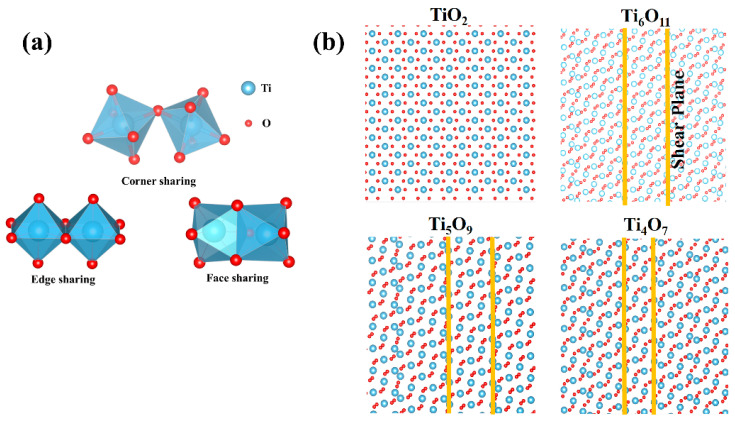
(**a**) Coordination modes of TiO_6_ octahedra: corner-sharing, edge-sharing, face-sharing. (**b**) The crystal presents a periodic layered stacking: alternating *n* layers of complete TiO_2_ units and 1 layer of anoxic TiO layers.

The crystal structure of Magnéli-phase titanium oxides is derived from the rutile-type TiO_2_ lattice, with oxygen octahedra coordinated around titanium atoms as the fundamental building blocks. As shown in Figure 2a, these TiO_6_ octahedra extend in three-dimensional space through corner-sharing or edge-sharing configurations. Crystallographic analysis revealed that the unit cell composition of the Ti_n_O_2n−1_ can be rationalized as a combination of *n* intact TiO_2_ units and one oxygen-deficient TiO unit. This structural reconstruction is achieved through periodic planar oxygen vacancy defects—specifically, an oxygen vacancy plane is generated every *n* layers of rutile-type structural units. The ordered arrangement of these defects reduces the crystal symmetry from the parent tetragonal system (P4_2_/mnm) to triclinic (P $\overset{-}{1}$) [23,24,25]. The introduction of oxygen vacancies induces fundamental alterations in the polyhedral connectivity (Figure 2b). Within the defect layers, adjacent TiO_6_ octahedra couple via face-sharing mechanisms, forming crystallographic shear planes that structurally compensate for lattice distortion caused by oxygen substoichiometry. The periodic arrangement of these shear planes significantly reduces the bandgap (Eg~0.1 eV), while the enhanced carrier mobility (μ ≈ 10^−2^ cm^2^ V⁻^1^ s⁻^1^) contributes to high intrinsic conductivity (σ~10^3^ S/cm). With an increasing Magnéli index *n* (Ti_4_O_7_ → Ti_6_O_11_), the system demonstrates distinct structure–property evolution: the planar oxygen vacancy density decreases linearly from 0.25 O/nm^2^ (*n* = 4) to 0.17 O/nm^2^ (*n* = 6), accompanied by shear plane spacing expansion from 1.2 nm to 1.8 nm. This structural progression induces two competing effects: The carrier concentration undergoes decay with reduced oxygen defects, leading to a decrease in conductivity by approximately two orders of magnitude. Concurrently, the increased shear plane spacing effectively suppresses active oxygen migration in the lattice, enhancing chemical stability and improving durability in oxidative and corrosive environments.

**Figure 3:**
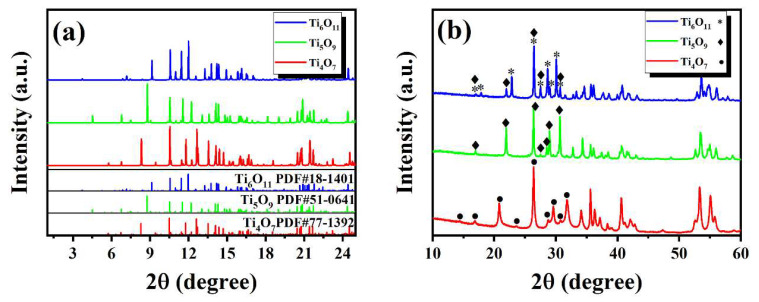
XRD patterns of Ti_n_O_2n−1_ samples as (**a**) initial powder (λ = 0.6199 Å) and (**b**) hot-press-sintered bulks (λ = 1.5169 Å).

As shown in Figure 3a, XRD characterization of the precursor Ti_n_O_2n−1_ (*n* = 4, 5, 6) powders revealed distinct Magnéli-phase features. The diffraction peaks of Ti_4_O_7_, Ti_5_O_9_, and Ti_6_O_11_ precisely matched their respective standard PDF cards, with no detectable impurity phases, confirming the high-purity single-phase nature of the TiO_2_-derived powders. In this study, we aimed to detect superlattice diffraction peaks (e.g., (121)_rutile_) along the 1/2 [011]_rutile_ crystallographic orientation using synchrotron radiation in order to validate the long-range ordered arrangement of oxygen vacancies within shear planes and provide direct evidence for the periodic crystallographic shear plane model in Ti_n_O_2n−1_ crystals [21,26,27]. However, under the current experimental conditions and sample characteristics, conclusive evidence of superlattice diffraction signals was not obtained due to the weak X-ray scattering of oxygen. Further characterization by conventional XRD of the hot-pressed bulk samples (Figure 3b) demonstrated that the Ti_4_O_7_ and Ti_5_O_9_ retained their single-phase structures, whereas the Ti_6_O_11_ bulk contained trace Ti_5_O_9_ impurities (>5 wt%). This phenomenon was attributed to excessive oxygen loss during hot-pressing under high-vacuum conditions (<10^−3^ Pa) with graphite dies, leading to partial reduction of Ti_6_O_11_ to Ti_5_O_9_. These results suggest the need for optimizing oxygen stoichiometry control through sintering atmosphere regulation (e.g., Ar/H_2_ mixtures) or interface passivation techniques.

### 3.2. Microstructure

**Figure 4:**
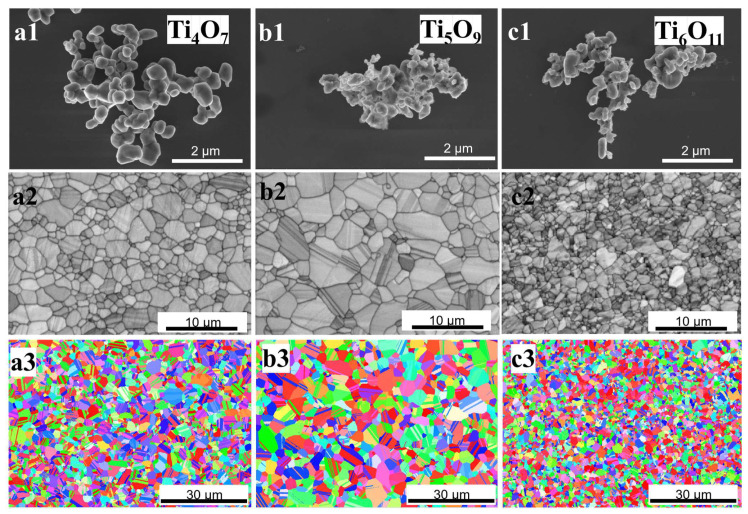
SEM images of Ti_n_O_2n−1_ samples as initial powder (**a1**–**c1**) and hot-press-sintered bulks (**a2**–**c2**), and EBSD IPF maps of the Ti_n_O_2n−1_ bulks (**a3**–**c3**).

As shown in Figure 4(a1–c1), the Magnéli-phase Ti_n_O_2n−1_ (*n* = 4, 5, 6) single-phase powders prepared using the carbothermal reduction method were all nano-sized. In order to systematically evaluate the thermoelectric properties of the Magnéli-phase structures, hot-press sintering was employed for powder densification. This process requires precise control to maintain single-phase integrity while achieving optimal densification. Previous investigations revealed that excessive densification temperatures lead to uncontrollable oxygen vacancy concentrations and secondary phase formation, while pressure optimization critically determines the final density. These combined factors significantly influence the accuracy of electrical and thermal conductivity measurements, ultimately affecting the reliability of thermoelectric performance characterization. SEM analysis (Figure 4(a2–c2)) demonstrated defect-free surfaces without observable voids, confirming excellent densification and structural homogeneity. The Archimedes method measurements (Table 1) corroborated these findings, showing relative densities exceeding 97% for all bulk materials. Electron backscatter diffraction (EBSD)-based grain size analysis (Figure 4(a3–c3)) revealed average dimensions of 2.4 (±0.1) μm for Ti_4_O_7_, 3.8 (±0.1) μm for Ti_5_O_9_, and 2.0 (±0.1) μm for Ti_6_O_11_. Notably, EBSD pole figure analysis confirmed the absence of a crystallographic texture in all three materials, providing direct evidence of isotropic grain growth mechanisms during the hot-press sintering process.

### 3.3. Electrical Properties

**Figure 5:**
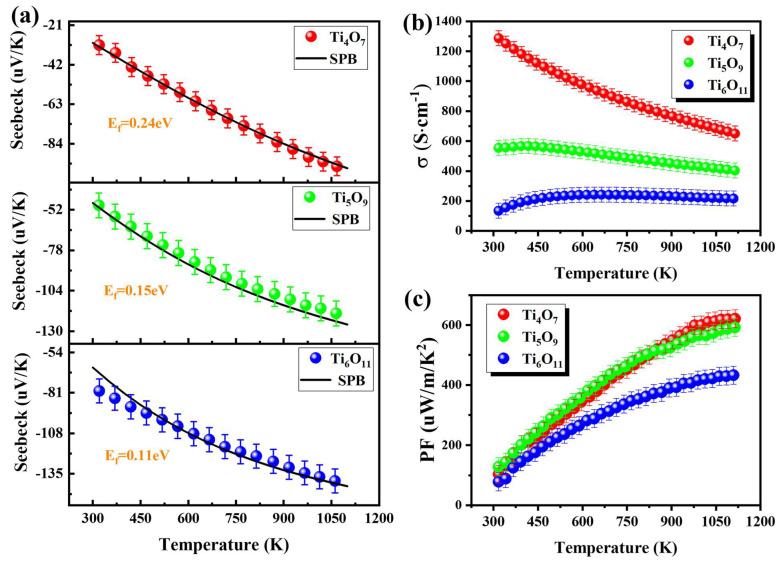
Temperature dependence of the Seebeck coefficient (spheres are test results; black solid line lines are the SPB model fits) (**a**), electrical conductivity (**b**), and thermoelectric power factor (**c**) for Ti_n_O_2n−1_.

As shown in Figure 5a, all Magnéli phases exhibited negative Seebeck coefficients (S = −30~−135 μV·K⁻¹), confirming the *n*-type semiconductor transport mechanism. The absolute value of the Seebeck coefficient increased as the oxygen content increased over the whole temperature range investigated, and it increased with the temperature for Ti_n_O_2n−1_ (*n* = 4, 5, 6). We used the single parabolic band model combined with the Seebeck coefficient inversion technique to obtain the represented reduced Fermi (η) and Fermi level (E_f_) [30]. Based on the Boltzmann transport theory, the following expressions were derived:

(1) $$S=\frac{k_{B}}{e}(\frac{\left(\frac{5}{2}+\lambda \right)F_{\frac{3}{2+\lambda }}}{\left(\frac{3}{2}+\lambda \right)F_{\frac{1}{2+\lambda }}}-\eta )$$

With the scattering parameter λ = −1/2 (acoustic phonon scattering dominance), Equation (1) simplifies to:

(2) $$S=\frac{k_{B}}{e}\left(\frac{2F_{1}}{F_{0}}-\eta \right)$$

where k_B_ is Boltzmann’s constant, e is the elementary charge, and division of the two gives a constant of 86.1733 μV/K, where η represents the reduced Fermi level:

(3) $$\eta =\frac{E_{f}}{k_{B}T}$$

F_i_ denotes the Fermi–Dirac integrals:

(4) $$F_{i}\left(\eta \right)=\int_{0}^{\infty }f\varepsilon^{i}d\varepsilon =\int_{0}^{\infty }\frac{\varepsilon^{i}}{1+\exp(\varepsilon -\eta )}d\varepsilon$$

**Figure 6:**
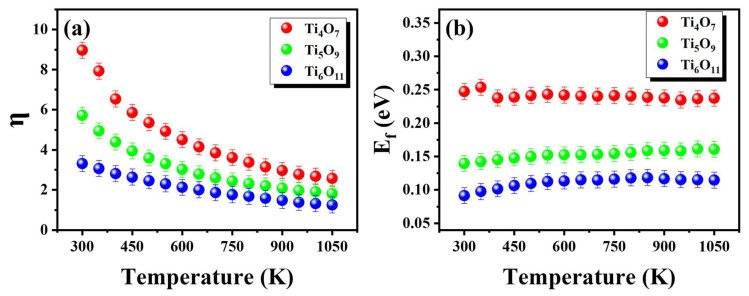
Relationship between (**a**) η, (**b**) E_f_, and temperature in the SPB model.

Through the experimental Seebeck coefficient inversion for η determination (Figure 6a), subsequent calculations of E_f_ via Equation (3) were carried out (Figure 6b). The E_f_ values for the Magnéli phases were averaged separately, yielding values of 0.24 eV for Ti_4_O_7_, 0.15 eV for Ti_5_O_9_, and 0.11 eV for Ti_6_O_11_. The black solid line in Figure 5a shows the fitted curves obtained from the SPB model, indicating that the SPB model is highly effective for evaluating thermoelectric properties.

The temperature-dependent conductivity in Figure 5b reveals that Ti_4_O_7_ possessed the highest intrinsic conductivity (~1287 S·cm^−1^) at room temperature, outperforming Ti_5_O_9_ (558 S·cm^−1^) and Ti_6_O_11_ (134 S·cm^−1^) while maintaining a five-order advantage over pristine TiO_2_ (σ < 10^−3^ S·cm^−1^) [11]. Over the whole temperature range investigated, Ti_4_O_7_ showed negative temperature coefficient behavior, indicating metalloid-like conduction [31]; Ti_5_O_9_ maintained quasi-steady conductivity (Δσ < 5%) below 450 K but exhibited a metal-like thermal response at medium–high temperatures (T > 450 K); and Ti_6_O_11_ followed typical semiconductor behavior, with increasing conductivity [32]. This divergence originated from structural differences: The density of oxygen-vacancy-induced face-sharing TiO_6_ octahedral shear planes decreased with increasing the *n*-value. The high-density shear planes in the Ti_4_O_7_ enhanced electron delocalization, resulting in superior conductivity, but they compromised the absolute Seebeck coefficient (|S| < 30 μV·K^−1^). The power factor (PF = S^2^σ) evolution in Figure 5c shows that the Ti_4_O_7_ and Ti_5_O_9_ reached peak PF values of 618 μW·m^−1^·K^−2^ and 591 μW·m^−1^·K^−2^ at T = 1100 K (error overlapping bars), representing 43% and 36% enhancements over Ti_6_O_11_ (432 μW·m^−1^·K^−2^). This suggests that moderate modulation of the oxygen vacancy concentration (*n* = 4 ⟶ 5) can optimize the carrier effective mass while preserving high conductivity, leading to significant breakthroughs in thermoelectric performance at the Pareto frontier.

### 3.4. Thermal Conductivity

**Figure 7:**
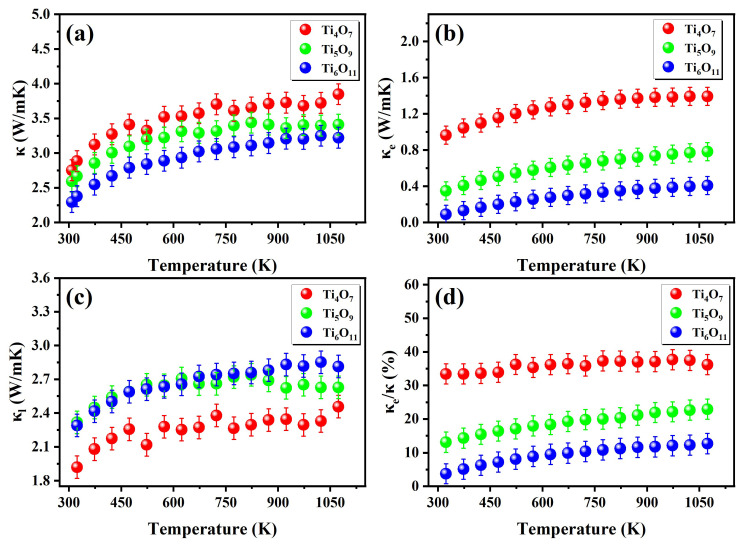
Thermal conductivity as a function of temperature for Ti_n_O_2n−1_: (**a**) total thermal conductivity κ, (**b**) electronic thermal conductivity κ_e_, and (**c**) lattice thermal conductivity κ_e_, (**d**) as a percentage of κ_e_/κ.

As shown in Figure 7a, the total thermal conductivity (K = κe + κl) of the Magnéli-phase Ti_n_O_2n−1_ systems exhibited a systematic decrease with increasing the *n*-value, which aligns well with previous studies [33,34]. In the temperature range of RT-1100 K, the measured total thermal conductivities for Ti_4_O_7_, Ti_5_O_9_, and Ti_6_O_11_ were 2.7–3.8 W·m^−1^·K^−1^, 2.6–3.4 W·m^−1^·K^−1^, and 2.3–3.4 W·m^−1^·K^−1^, respectively. The lower value of the thermal conductivity of the hot-pressed Magnéli-phase Ti_n_O_2n−1_ compared to the rutile single-crystal TiO_2_ (6–8 Wm/K) [35] was attributed to phonon scattering at grain boundaries. The increasing oxygen vacancy concentration induced two regulatory mechanisms on the thermal transport properties: the enhanced carrier concentration significantly improved the electronic thermal conductivity (Figure 7b), while shear-plane induced lattice distortion intensified the phonon scattering, thereby suppressing the lattice thermal conductivity. To quantitatively analyze the electron–phonon coupling mechanism, we employed the Wiedemann–Franz approximation to establish an electronic thermal conductivity model (Equation (5)), with electronic and lattice thermal conductivity derived through differential analysis.

(5) $$\kappa_{e}=L\sigma T$$

where σ denotes the electrical conductivity, T is the absolute temperature, and L represents the Lorenz number. Considering theoretical discrepancies between degenerate (2.45 × 10^−8^ W·Ω·K^−2^) and non-degenerate limits (1.5 × 10^−8^ W·Ω·K^−2^), we obtained a precise computational framework for L-values using a single parabolic band model combined with the Seebeck coefficient inversion technique [30]. Based on the Boltzmann transport theory (Equation (1)), the following expressions were derived:

(6) $$L=\frac{k_{B}^{2}}{e^{2}}\frac{\left(\frac{3}{2}+\lambda \right)\left(\frac{7}{2}+\lambda \right)F_{\frac{1}{2+\lambda }}F_{\frac{5}{2+\lambda }}-{\left(\frac{5}{2+\lambda }\right)}^{2}F_{\frac{3}{2+\lambda }}^{2}}{{\left(\frac{3}{2+\lambda }\right)}^{2}F_{\frac{1}{2+\lambda }}^{2}}$$

With a scattering parameter of λ = −1/2, Equation (6) simplifies to:

(7) $$L=\frac{k_{B}^{2}}{e^{2}}\frac{3F_{0}F_{2}-4F_{1}^{2}}{F_{0}^{2}}$$

Through experimental Seebeck coefficient inversion for η determination, subsequent calculations of L via Equations (2) and (4) enabled the precise determination of κ_e_ using Equation (5).

Figure 7b reveals a strong positive correlation between κ_e_ and the electrical conductivity in the Magnéli phases. At room temperature, the κ_e_ contributions to the total thermal conductivity were 33.4%, 13.1%, and 3.8% for Ti_4_O_7_, Ti_5_O_9_, and Ti_6_O_11_, respectively (as shown in Figure 5c). These results demonstrate distinct thermal transport regimes: (1) electron-dominated transport in high-conductivity Ti_4_O_7_, (2) electron–phonon mixed conduction in Ti_5_O_9_, and (3) phonon-dominated thermal transport in Ti_6_O_11_. Theoretically, the lattice thermal conductivity should exhibit an inverse correlation with the shear plane density (reciprocal of shear plane spacing) due to enhanced phonon scattering at the shear planes. However, the experimental results show nearly identical κ_l_ values between Ti_6_O_11_ and Ti_5_O_9_ (Δκ_l_ < 0.2 W·m^−1^·K^−1^) [25], which can be attributed to two concurrent mechanisms: phase purity effects and grain size effects. XRD analysis revealed that the Ti_6_O_11_ bulk contained above 5 wt% Ti_5_O_9_ as a secondary phase (Figure 3b). The lower shear plane density (*n* = 5) of this impurity phase likely counteracted the expected κ_l_ reduction. We attempted to achieve single-phase Magnéli structures under different sintering temperatures, resulting in a decreased average grain size from 3.8 μm to 2.0 μm (Figure 4(b2,c2)). According to the Callaway model, larger grains extend the phonon mean free path, systematically elevating κ_l_.

**Figure 8:**
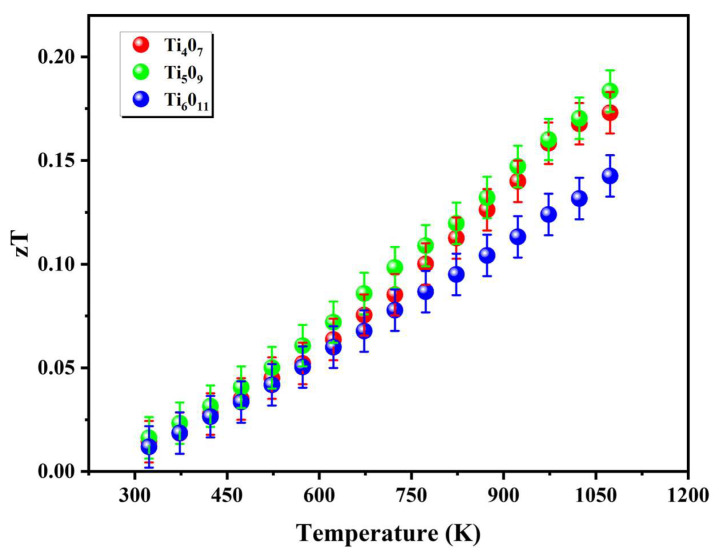
Figure of merit (zT) as a function of temperature.

Systematic analysis based on experimental data revealed that the evolution of the thermoelectric figure of merit in the Magnéli-phase titanium oxides (TiₙO_2n−1_) exhibited significant temperature dependence but a non-monotonic correlation with the *n*-value (Figure 8). Notably, the zT trajectories of the Ti_4_O_7_ and Ti_5_O_9_ showed substantial overlap within the RT-1100 K range, suggesting potential similarities in their electron–phonon coupling mechanisms. Specifically, the zT value of the Ti_4_O_7_ increased linearly from 0.015 at room temperature to 0.17 at 1100 K, with performance limitations primarily attributed to the dominant contribution of electronic thermal conductivity (κ_e_/κ > 33%), closely linked to its metallic-like electronic structure and strong electron–phonon scattering. When *n* increased to 5, the reduced oxygen vacancy concentration in the Ti_5_O_9_ lowers its electronic thermal conductivity to κ_e_/κ = 13–22%. Through synergistic optimization of the carrier concentration and Seebeck coefficient, the Ti_5_O_9_ achieved a zT value of 0.18 at 1100 K. In contrast, the Ti_6_O_11_ exhibited the lowest total thermal conductivity (κ~2.3 W·m^−1^·K^−1^), and its diminished electrical conductivity resulted in an insufficient power factor, ultimately restricting its zT value to 0.14 at 1100 K. This phenomenon highlights an inherent competition between thermal conductivity reduction and electrical transport maintenance in Magnéli-phase systems, necessitating atomic-scale defect engineering to achieve the cooperative optimization of both properties.

## 4. Conclusions

In this work, high-density (>97% relative density) single-phase Magnéli-phase titanium oxide (TiₙO_2n−1_, *n* = 4–6) bulk materials were successfully synthesized via carbothermal reduction combined with hot-pressing sintering. The structure–property relationships among the phase evolution, microstructural characteristics, and thermoelectric performance were systematically investigated. The absolute Seebeck coefficient decreased with increasing the oxygen vacancy concentration (lower *n*-values). Both the electrical conductivity and total thermal conductivity were negatively correlated with the oxygen vacancy concentration. Ti_4_O_7_ demonstrated κ = 3.2 W·m^−1^·K^−1^ with a dominant electronic contribution (κ_e_/κ = 33%), whereas Ti_5_O_9_ exhibited a reduced κ = 2.8 W·m^−1^·K^−1^ (κ_e_/κ = 13–22%). The thermoelectric figure of merit (zT) showed pronounced temperature dependence but a non-monotonic correlation with the *n*-values. At 1100 K, the peak zT values reached 0.17, 0.18, and 0.14 for Ti_4_O_7_, Ti_5_O_9_, and Ti_6_O_11_, respectively. The performance limitation of Ti_4_O_7_ stemmed from strong electron–phonon scattering (Lorenz number L = 2.1 × 10^−8^ W·Ω·K^−2^) induced by its metallic-like electronic structure (high density of states near the Fermi level). In contrast, Ti_5_O_9_ achieved superior high-temperature zT stability (>900 K) through optimized oxygen vacancy concentration and enhanced carrier mobility. Our study provides critical experimental insights for designing high-performance oxide thermoelectric materials via oxygen vacancy ordering and stoichiometric control. In addition, future investigations could systematically explore advanced doping strategies and nano-structuring to optimize the carrier concentration and phonon scattering synergistically, thereby pushing the thermoelectric figure of merit (zT) beyond current limitations.

## Figures and Tables

**Table 1:** Grain size, density, relative density, and conductivity of the Ti_4_O_7_, Ti_5_O_9_, and Ti_6_O_11_ at room temperature.

Sample	Ti_4_O_7_	Ti_5_O_9_	Ti_6_O_11_
grain size, μm	2.4 (1)	3.8 (1)	2.0 (1)
Density, g/cm^3^ (theory)	4.238	4.2829	4.295
Density, g/cm^3^ (measured)	4.231	4.228	4.184
Relative density, %	99.8	98.7	97.4
Conductivity, S cm^−1^ (measured)	1287	554	137
Conductivity, S cm^−1^ (literature)	992 [21] 1252 [28]	631 [29]	63 [29]

## Data Availability

The original contributions presented in this study are included in the article. Further inquiries can be directed to the corresponding author(s).
